# Robust algorithm for arrhythmia classification in ECG using extreme learning machine

**DOI:** 10.1186/1475-925X-8-31

**Published:** 2009-10-28

**Authors:** Jinkwon Kim, Hang Sik Shin, Kwangsoo Shin, Myoungho Lee

**Affiliations:** 1 Department of Electronic and Electrical Engineering, Yonsei University, Seoul, Korea

## Abstract

**Background:**

Recently, extensive studies have been carried out on arrhythmia classification algorithms using artificial intelligence pattern recognition methods such as neural network. To improve practicality, many studies have focused on learning speed and the accuracy of neural networks. However, algorithms based on neural networks still have some problems concerning practical application, such as slow learning speeds and unstable performance caused by local minima.

**Methods:**

In this paper we propose a novel arrhythmia classification algorithm which has a fast learning speed and high accuracy, and uses Morphology Filtering, Principal Component Analysis and Extreme Learning Machine (ELM). The proposed algorithm can classify six beat types: normal beat, left bundle branch block, right bundle branch block, premature ventricular contraction, atrial premature beat, and paced beat.

**Results:**

The experimental results of the entire MIT-BIH arrhythmia database demonstrate that the performances of the proposed algorithm are 98.00% in terms of average sensitivity, 97.95% in terms of average specificity, and 98.72% in terms of average accuracy. These accuracy levels are higher than or comparable with those of existing methods. We make a comparative study of algorithm using an ELM, back propagation neural network (BPNN), radial basis function network (RBFN), or support vector machine (SVM). Concerning the aspect of learning time, the proposed algorithm using ELM is about 290, 70, and 3 times faster than an algorithm using a BPNN, RBFN, and SVM, respectively.

**Conclusion:**

The proposed algorithm shows effective accuracy performance with a short learning time. In addition we ascertained the robustness of the proposed algorithm by evaluating the entire MIT-BIH arrhythmia database.

## Background

Arrhythmia is a form of heart conduction system disease that causes an inefficient heart beat. Typically, arrhythmia is diagnosed through an electrocardiogram procedure. Because arrhythmia represents abrupt and abnormal ECG beats, physicians diagnose arrhythmia based on long-term ECG data using an ECG recording system like the Holter recorder. In addition, various remote and mobile healthcare systems that are adapting ECG recorders are increasing in number these days, and the importance of an automatic arrhythmia classification algorithm is being increasingly recognized. There are many existing studies on the classification of arrhythmia, and the algorithm is generally composed of the pre-processing part, the feature extraction part, and the classification part.

The pre-processing part removes noise components and does other forms of processing for more accurate feature extraction or classification. The main noise components of ECG include baseline drift, power line interference, and moving artefacts [1]. Various reports have been published on the filtering methods for removing noise components while preserving both ECG morphology and fast processing.

The feature extraction part makes feature vectors that are used later in the classification part. Various signal compression algorithms are used to represent the signal's characteristics efficiently with a small computational burden. Many transformation methods, such as principal component analysis (PCA), or independent component analysis (ICA)[2], are very frequently used. The classification part makes an arrhythmia diagnosis using acquired feature vectors from the feature extraction part. Statistical approaches [3], fuzzy inference approaches, and neural network approaches [4] are the typically used methods for ECG pattern classification. The statistical approaches classify ECG patterns by using statistical modelling, which is acquired from the data. However, in the case of statistical approaches, there are some difficulties in acquiring many types of data and in selecting a model that best represents data distribution. The fuzzy inference approaches have less computational burden than others, but those approaches have a subjective nature because membership function selection is accomplished by applying the opinions of experts and repeated experimentation. In addition, the complex decision region acquired through the machine learning approach is considered as one of the neural network approaches. However, a huge learning dataset, large computational burden and extended learning time are pointed out as the main shortcomings of the neural network approaches.

There have been many studies that found ways to overcome the weaknesses of the neural network approaches. Most of the recent research projects on improving the arrhythmia classification algorithm are classified into two types of approaches. One approach involves finding a better ECG feature extraction method, such as dimension reduction, and the other is concerned with finding a better classifier. Many studies on ECG feature extraction have been reported using PCA and ICA for dimension reduction; and Fourier transform [5] and Wavelet transform [4,6] for frequency component representation. There are also many studies that adapted a new classifier such as a Support Vector Machine (SVM)[7]. Some comparative studies of various data reduction[8-10], feature extraction[10,11], and classification methods[11] were presented recently, but the size of test data set was relatively small.

As a kind of classifier, Extreme Learning Machine (ELM) was able to overcome the difficulties of a neural network through a fast learning speed and high performance [12]; the problems that persisted were an extended learning time for a gradient-based learning algorithm, the possibility of local minima converging, and a degraded performance due to overtraining. A BPNN that is typically used as a learning algorithm on a Single Hidden Layer Feedforward Neural Network (SLFN) adjusts the weights between layers based on propagating errors from the output layer to the input layer. However, it was demonstrated that a SLFN classifier that has randomly selected weights between the input layer and the hidden layer may have the ability to classify the data set only with the controlling weights between the hidden layer and the output layer [13]. Based on this research result, the ELM acquired optimal weights between the hidden layer and the output layer analytically, with randomly selected weights between the input layer and the hidden layer.

The performance of the classification not only depends on the classifier, but also depends on the features, and better ECG signal processing is of great benefit to feature extraction. Thus in this study, we proposed and analyzed the proper signal processing methods for each part of arrhythmia classification algorithm: preprocessing, feature extraction, and classification part, in an effort to develop robust algorithm. We used Morphology Filter (MF) as the pre-processing part to remove the noise component while preserving ECG morphology, and time domain features and morphology features of ECG compressed by PCA as the feature extraction part. For the classification part we used ELM in order to reduce learning time while maintaining high accuracy. We first evaluated the each component of the proposed arrhythmia classification algorithm separately, and made the comparative study of the performances of the algorithm using an ELM, NN, RBFN, or SVM as the classifier. In addition, the performance of proposed algorithm was compared with that of other researches.

## Methods

**Figure 1:**
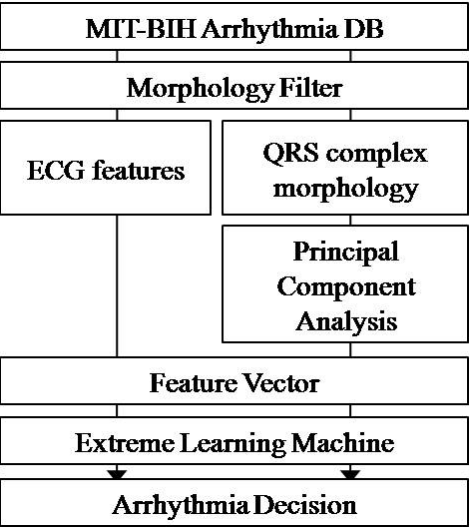
**Block diagram of the proposed algorithm**.

The composition of the proposed algorithm is shown in Figure 1. This paper presents MF based pre-processing to remove high frequency noise components and baseline drift, as well as to preserve ECG morphology. MF is a type of filtering method which has the virtue of preserving the sharpness of the QRS complex, and this has important meaning for ECG. The feature vectors are made of several descriptive parameters and compressed 250 ms QRS complex morphology data by the PCA from a given ECG signal after the MF stage. Then, ELM is trained and classified using these feature vectors. In the evaluation stage we used the entire MIT-BIH arrhythmia database to maintain the robustness of the proposed algorithm.

### Morphology Filtering

It was proposed a method of ECG morphological filtering which filters baseline drift and high frequency ECG noise with less distortion than in the original ECG signal, and with less computational burden [14]. *f*_0 _is the ECG signal containing noise components, and the ECG signal without baseline drift, *f*_bc_, is acquired through Equation (1).(1)

In Equation (1), '°' and '·' represent the opening operation and the closing operation, respectively. *B*_0 _and *B*_c _are structural elements which have zero amplitude sequence, and are 0.2 and 0.3 times the length of the sampling frequency *F*_s _In the case of high frequency noise, the filtered signal is defined as Equation (2).(2)

where '⊕ ' and '⊖ ', dilation and erosion respectively, are morphological operators. *B*_2 _is the structural element [0,0,0,0,0] to remove the high frequency noise. And, *B*_1_, which is a structural element [0,1,5,1,0], compensates for the high frequency components of the original signal, which are removed by *B*_2_

### Principal Component Analysis

As the most representative dimension reduction technique, PCA projects multidimensional data onto space which is constructed of axes in the order of distribution. Thus, the given data can be represented by fewer dimensions in terms of the minimum mean square error. When *d *dimensional original data projects onto *d*' dimensional space, the error is represented by Equation (3), and the equation is referred to as the criterion function.(3)

As Equation (3) is the sum of the mean square error, we can obtain optimal basis vectors by minimizing Equation (3). And, by using a scatter matrix ***S***, the vectors ***e***_*i *_minimizing Equation (3) are calculated by Lagrange multiplier. As a result of the minimization of Equation (3), we take the Equation (4) using the Lagrange multiplier *λ*_*i*_.(4)

The bases from Equation (4) are eigenvectors of the scatter matrix. To maximize distribution of projected data in *d*' dimension, we have to select *d*' bases from Equation (4) in the order of their eigenvalues. The scatter matrix ***S ***is real and symmetric, thus the eigenvectors ***e***_*i *_are mutually orthogonal. The reduced dimensional space is made up of selected eigenvectors.

### Extreme Learning Machine

ELM makes up for the defects of gradient-based learning algorithm by analytically calculating the optimal weights of the SLFN. First, weights between the input layer and the hidden layer are randomly selected to make the SLFN into a linear equation, and then we can obtain the optimal value for weights between the output layer and the hidden layer by calculating the linear matrix equation. When we have *N *samples of input data and  hidden neurons, the SLFN neural network is defined as Equation (5).(5)

where ***w***_*i *_is the weight vector between the *i*th neuron in the hidden layer and the input layer, *b*_*i *_means the bias of the *i*th neuron in the hidden layer; *x*_*i *_is the *j*th input data vector; *g*(.) is an active function of the hidden neuron; ***β***_*i *_is the weight vector between the *i*th hidden neuron and the output layer; and ***t***_*j *_means the target vector of the *j*th input data. When ***w***_*i *_and *b*_*i *_are constant, *g*(***w***_*i*_·***x***_*j*_+*b*_*i*_) in Equation (5) which is the output of the *i*th hidden neuron, can be a constant matrix according to ***x***_*i *_Thus, Equation (5) can be reformulated as a matrix equation to form Equation (6) by using that output matrix of the hidden layer ***H***.(6)

In Equation (6), the target vector ***T ***and the output matrix of the hidden layer ***H ***comprise a linear system. Thus, the learning procedure of the neural network becomes finding the optimal weight matrix ***β ***between the output layer and the hidden layer. This process can be accomplished using the Moor-Penrose Generalized Inverse of ***H***, as is shown in Equation (7).(7)

By using the Moor-Penrose Generalized Inverse of ***H***, the optimal ***β ***has the minimum norm least-squares solution of the linear system. With this process we can establish two effects. The first effect is that we can take a minimum error condition, because obtained  is least-squares solution. In addition, optimal  is not only the least-squares solution but also the minimum norm among these solution. Thus, ELM has a better generalization performance than a typical BPNN [15].

### Back Propagation Neural Network

BPNN is a learning algorithm for the multi-layer neural network which adjusts the weights and the biases by propagating the errors from the output layer to the input layer[16]. The criterion function of the BPNN is expressed as Equation (8):(8)

where ***w***, ***t***_*k*_, and ***z***_*k *_are the weight vector, the target vector, and the output vector, respectively. BPNN is the representative gradient descent method for searching the weight vectors ***w***, which are initialized with arbitrary value at the beginning, and then are adjusted according to the most rapid decrease of *J*(***w***).(9)

BPNN takes a long time to learn and has the risk of falling into a local minimum, because it solves the Equation (9) iteratively until *J*(***w***) reaches the minimum.

### Radial Basis Function Network

RBFN is a kind of neural network whose hidden neurons have a RBF activation function. RBF is a function which depends only on the radial distance from a vector[16]. The RBFN performs the nonlinear transformation from the input feature space, where the input patterns are not linearly separable, to the hidden unit space, where the transformed input patterns may be linearly separable. RBFN is expressed as Equation (10):(10)

where ***w***, ***φ***(·), and ***c***_*k *_are the weight vector between the hidden neuron and the output layer, the activation function, and the center of the *k*th activation function, respectively. And ***x ***and ***y ***are the input and output vector. Typically, the activation function uses the gaussian function or other bell-shaped functions. Thus, we had 3 kinds of parameters to accomplish the learning process with the given architecture, those are ***w***, ***c***_*k*_, and the spreads of RBF. In this paper, ***c***_*k *_were acquired through k-means clustering, and the spreads of RBF were chosen by the normalization as Equation (11).(11)

where *d*_*max *_is the maximum distance between any 2 centers, and *m *is the number of centers. Finally, ***w ***are computed by means of the pseudo-inverse method.

### Support Vector Machine

SVM aims to achieve structural risk minimization using the concept of margin unlike criterion functions of other pattern recognition algorithms of which the goal is minimization of empirical error[17]. SVM extends the applicability of linear classifier to non-linear separable data by using the kernel method. Non-linear SVM is considered to solve a conditional optimization problem as Equation (12):(12)

where *K*(***A, B***) is a kernel function of ***A ***and ***B***, *α *means the Lagrange multiplier, *t *has 1 or -1 as target value, *C *is the weight factor between minimizing the error and maximizing the margin. Thus when using SVM, there are two parameters to be decided, *K*(***A, B***) and *C*. In this paper, we used a radial basis function kernel, like Equation (13), as a kernel function and found *C *through a simulation.(13)

SVM is basically a binary classifier. But in this paper, we used a one-against-all multiclass SVM because of many classes for each arrhythmia.

### Experimental Method

The proposed algorithm was evaluated using the MIT-BIH arrhythmia database [18]. The evaluation with the entire MIT-BIH arrhythmia database shows the robustness of proposed algorithm. (102, 104, and 114 ECG data files in the MIT-BIH arrhythmia database were excluded since those files are not recorded on Modified Lead II.) We selected major beat types with a coverage ratio exceeding 1% in the entire MIT-BIH arrhythmia database. By doing that, we had 6 major beat types. They are normal beats, left bundle branch block (LBBB) beats, right bundle branch block (RBBB) beats, premature ventricular contractions (PVC), atrial premature beats (APB), and paced beats (PB). The total of the coverage ratios of those 6 major beat types is 97.76% as is shown in Table 1.

After 6 beat types were extracted from the entire MIT-BIH database, a randomly chosen quarter of database was used as the training dataset, and the other three-fourths were used as the test dataset. 4-fold cross-validation[19] was used to estimate the classification accuracy. We used a full three-fourths of the database as the testing dataset, but in the case of the training dataset, we randomly selected 5000 beats from the normal beats and 1000 beats from the other beat types, in order to prevent underestimation of the abnormal beat types. The composition of the training dataset and the test dataset is shown in Table 1.

**Table 1:** Composition of the data set

Beat type	Number of beats in Test data set	Number of beats in Training data set	Possession rate (%)
Normal	54516	5000	70.2573
LBBB	6036	1000	7.7746
RBBB	5410	1000	6.9889
PVC	5293	1000	6.8166
APB	1896	1000	2.4407
PB	2702	1000	3.4853
Total	75853	10000	97.76

Possession rate means that possession ratio of each beat type in the entire MIT-BIH arrhythmia data base. LBBB: left bundle branch block, RBBB: right bundle branch block, PVC: premature ventricular contraction, APB: atrial premature beat, PB: paced beat

The constructed ECG dataset was filtered by a MF. Then, the current RR interval (RRI), the ratio of the current RRI against the next RRI (RRIR), the ratio of the current RRI against the average of the latest 10 beats (10RRIR), and the R peak amplitude (Ramp) were extracted from the filtered signal as ECG descriptive features. The RRIR was introduced to reflect the compensatory pause of the APB and PVC, and the 10RRIR compensates for the variation in the normal RR interval range. Besides ECG descriptive features, morphology features were acquired from ECG data around an R peak (250 ms, 90 samples), which is provided by the databases' annotation. The morphology features compressed by PCA and the descriptive features were included in the feature vectors to train and test ELM. The evaluation was performed with Matlab R2007b and with Intel Quad core 2.40 GHz, 2 GB RAM, Microsoft windows XP platform.

## Results

This section presents the results of the analysis of each component. Concerning the morphology filtering component, we compared MF with a typical filtering method in terms of preserving ECG morphology, and for the feature extraction component we analyzed the distribution of features in each beat type to estimate the efficiency of the features. Finally, for the classification component, we simulated the algorithm in terms of sensitivity, specificity, and the accuracy basis by varying the number of principal components and hidden neurons, in order to evaluate the optimal ELM structure. The obtained optimal results of the proposed algorithm were compared with those of other existing research papers in terms of the size of the training dataset, test dataset, and accuracy. The training time, which is another advantage of the proposed algorithm, was also evaluated with BPNN, RBF, or SVM based algorithm.

### Result of Morphology Filtering

**Figure 2:**
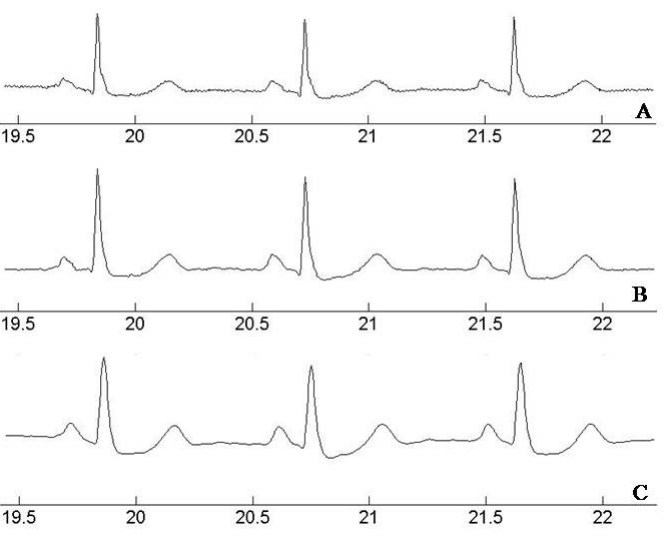
**Comparison of the result signals of Morphological filtering and general frequency based filtering**. (A) Contaminated ECG (101 file in MIT-BIH arrhythmia DB), (B) morphological filtered signal and (C) general filtered signal.

The advantages of MF is that it is able to filter noise components while preserving the original ECG morphology, and it has a small computational burden compared with typical frequency based filtering. The Figure 2 shows the comparison between the result signals of typical frequency based filtering [20] and that of MF on a 101 file in MIT-BIH arrhythmia DB. The typical frequency based filter composition clearly removed the high frequency noise component, but the high frequency components of QRS complex decreased while eliminating the EGM frequency band (generally 35 Hz). Thus, we can see a wider QRS complex in the result signal, in typical frequency based filtering, than the complex in an original ECG signal or a result MF signal. This characteristic creates possible confusion since ventricular arrhythmia beats generally have a wide QRS complex. As it can be seen in the result MF signal, this effect does not occur since MF is not established on frequency bases.

### Distribution of Feature Set Results

**Figure 3:**
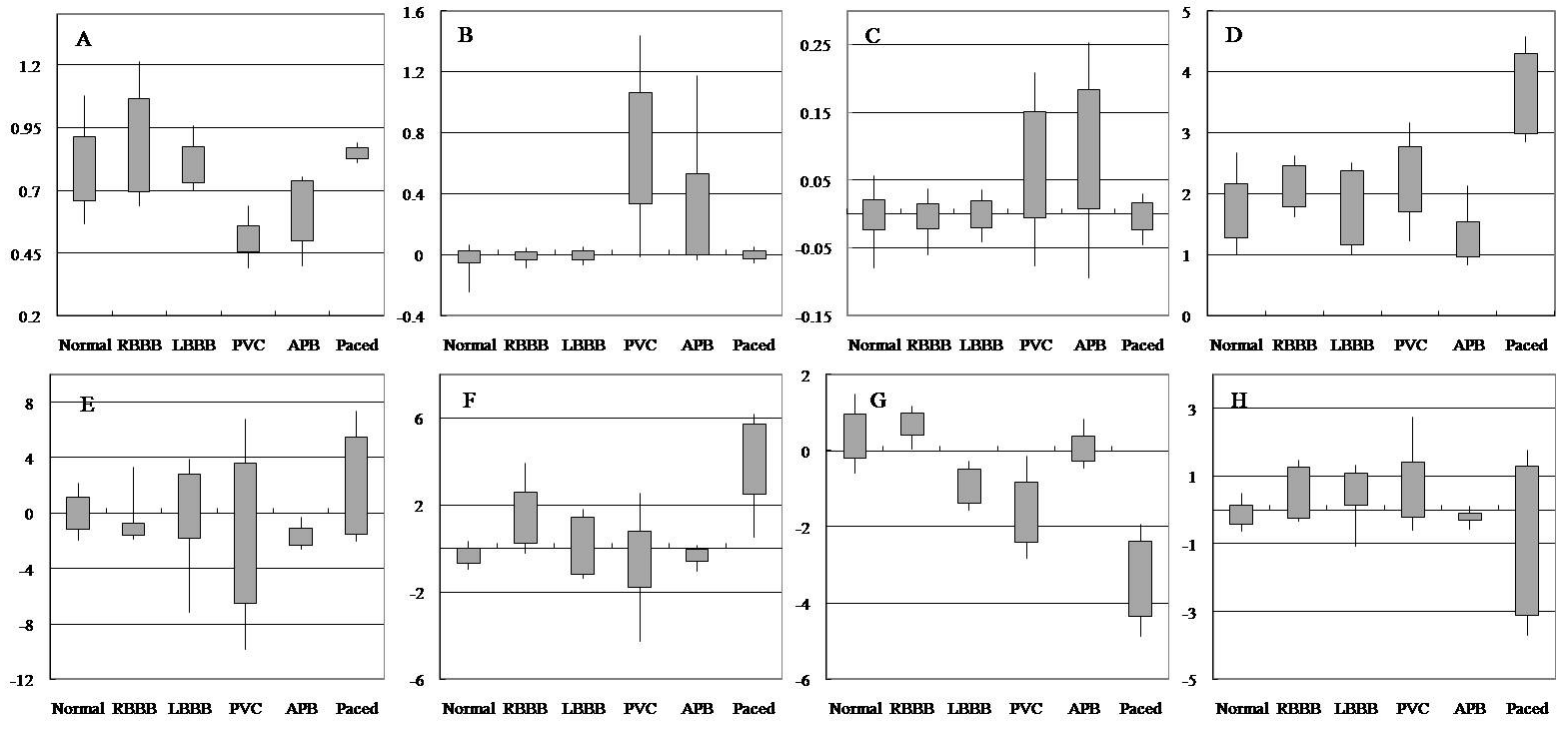
**The distribution of feature vectors**. The candle bar means the distribution of each beat type's feature. The uppermost part of the bar means 90% of the feature distribution level, the upper side of the box means 75%, the bottom side of the box means 25% and the bottommost part of the bar means 10%. (A) RRI: current RR interval, (B) RRIR: ratio of current RRI to next RRI, (C) 10RRIR: ratio of current RRI to average of late 10 beats, (D) Ramp: R peak amplitude, (E) 1st principal component, (F) 2nd principal component, (G) 3rd principal component and (H) 4th principal component.

The proposed algorithm used 4 ECG descriptive features. These are the RRI, the RRIR, the 10RRIR and Ramp. Also, the algorithm used 30 ECG morphological features compressed by PCA, which contained 99.99% of the total amount of morphology information. The Figure 3 shows the distribution of 4 ECG descriptive features and 4 principal components among the morphological features. In the cases of PVC and APB, the RRI, RRIR, and 10RRIR have very different distributions when compared to those of other beat types. They show reasonable distributions for premature beats such as PVC and APB. It seems that the RRIR is also desirable and reflects well on the compensation period. Concerning the distribution of the Ramp, PBs have remarkably larger amplitudes than other beat types. In the ECG beat type classification algorithm, having a feature that can separate other beats from normal beats is important since normal beats are 70% of the entire database. As all beat types have their largest displacement at the QRS complex, all beat types have similar distributions in the principal component1 (PC1). However, in PC2, the PB and RBBB are different from the normal beat. Also, in PC3, the LBBB, PVC and Paced beats are different from normal beats.

### Results of the Proposed Algorithm

The classifier of the proposed algorithm is ELM, which has the number of hidden neurons as the adjustable parameter affecting performance. In addition, in this application the number of principal components also affects performance. Thus, in order to analyze the optimal ELM structure, we evaluated the proposed algorithm with a varying number of hidden neurons and principal components. The criteria for evaluating the performance of the algorithm were sensitivity, specificity, and accuracy, and the formulas are as follows:

where TP, TN, FP, and FN are True Positive, True Negative, False Positive, and False Negative, respectively.

**Figure 4:**
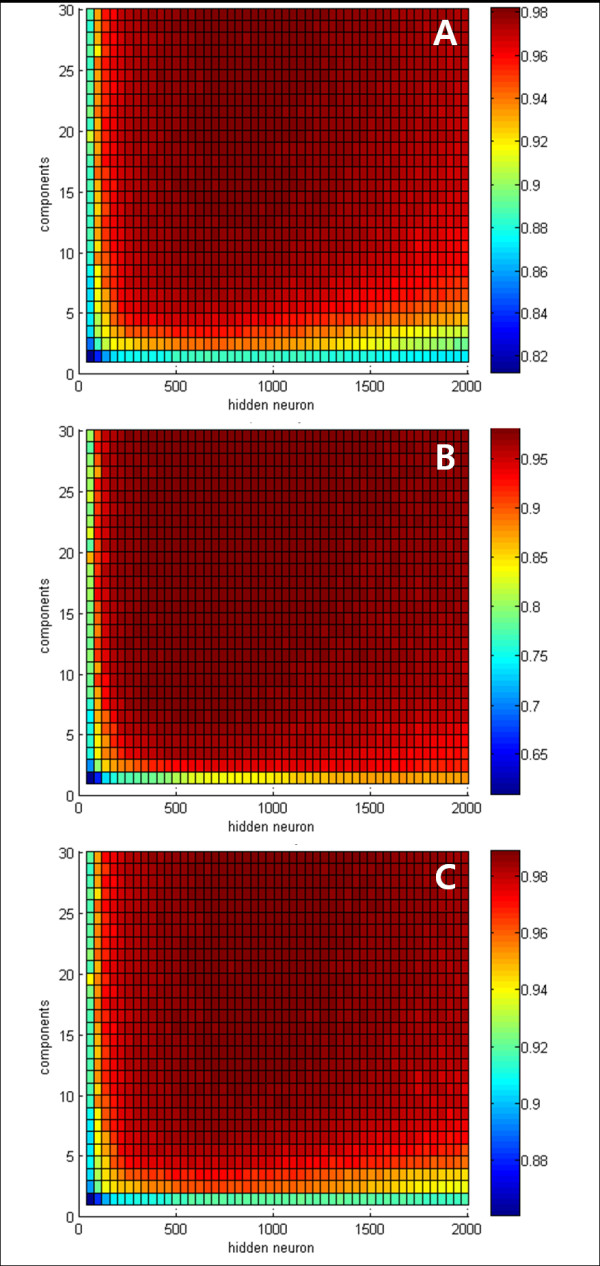
**Performance of the proposed algorithm**. (A) sensitivity, (B) specificity, and (C) accuracy.

The Figure 4 shows the results of each composition. In this study, we considered evaluating the proposed algorithm by changing the number of hidden neurons from 40 to 2000, and the number of principal components from 1 to 30. Performance improved as the number of hidden neurons and principal components in all three criteria areas increased. However, performance did not improve any more in the cases in which there were more than 10 principal components or 400 hidden neurons. In those cases using 720 hidden neurons and 14 principal components, the detailed beat classification results of the proposed algorithm are shown in Table 2. Whole beat types, except for APB and PVC, had greater than 98% sensitivity, but the sensitivity of PVC and APB was 90.36% and 89.24%, respectively.

**Table 2:** Results of the proposed algorithm with 720 hidden neurons and 14 components

Results from Proposed Algorithm	Actual heart beat type						
	
	Nor	LBBB	RBBB	PVC	APB	PB	Total
Normal	53885	90	35	276	188	1	54475
LBBB	83	5922	2	64	5	0	6076
RBBB	65	1	5356	29	5	0	5456
PVC	104	16	1	4783	6	0	4910
APB	379	7	16	141	1692	1	2236
PB	0	0	0	0	0	2700	2700
Total	54516	6036	5410	5293	1896	2702	75853

Accuracy, Sensitivity, Specificity (%)							

**Beat types**	**Nor**	**LBBB**	**RBBB**	**PVC**	**APB**	**PB**	**Avg.**

sensitivity	98.84	98.11	99.00	90.36	89.24	99.93	98.00
specificity	97.23	99.78	99.86	99.82	99.26	100.00	97.95
accuracy	98.39	99.65	99.80	99.16	99.01	100.00	98.72

The diagonal terms of the matrix are the number of beats correctly classified. LBBB: left bundle branch block, RBBB: right bundle branch block, PVC: premature ventricular contraction, APB: atrial premature beat, PB: paced beat

**Figure 5:**
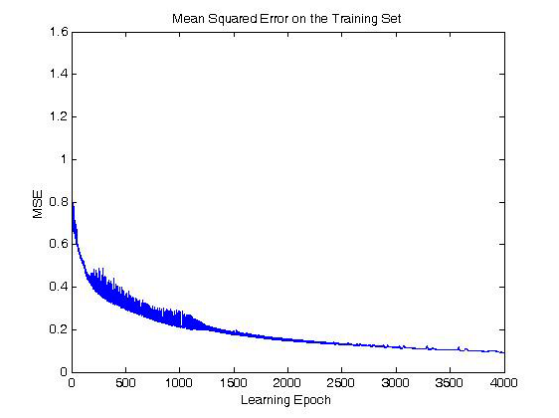
**The convergence curve of BPNN based algorithm with 0.002 in learning rate, 10 in hidden neurons, and 10 in principal components used**.

The results of the learning time are shown in Table 3, 4, 5, and 6. A comparative study was conducted to compare the learning time of the algorithms based on ELM with that of BPNN, RBF or SVM. The performance of BPNN is based on the learning rate, the momentum, the number of training iterations and hidden neurons, and the number of hidden layers used. In this simulation, the number of hidden layer was equal to that in the ELM case as 1, and the learning rate, iteration number, and a number of hidden neurons were used in the best result case. The simulation was evaluated by varying the learning rate from 0.0005 to 0.002. When the learning rate was either under 0.0005 or over 0.002, the accuracy of the algorithm couldn't converge on over 90%. And we also changed the number of hidden neurons from 10 to 100 with 10 steps. The performance of the algorithm using BPNN showed distinct decline when using over 50~80 hidden neurons. The termination point of the learning process was when the training accuracy converged because the variation of the error was very small (When the variation of the mean squared error between the target values and the values of the output neuron in the consecutive epochs is less than 10^-8^.) or the iteration number is over 4000. The momentum was fixed as 1. The Figure 5 shows the convergence curve of the BPNN based algorithm in the best result case, when the algorithm used the 10 hidden neurons with 0.002 as the learning rate and 4000 as the iteration number.

**Table 3:** Evaluation of the learning times and the testing accuracies among algorithms using ELM with 10 principle components

The number of hidden neurons	200	400	600	800	1000	1200	1400	1600	1800	2000
Training time (seconds)	0.95	3.13	7.09	**13.61**	22.48	35.13	50.47	70.11	94.30	123.77
Testing accuracy (%)	97.76	98.50	98.60	**98.72**	98.72	98.60	98.37	98.19	98.06	97.75

The bold column represents the structure of the network which shows the best accuracy.

**Table 4:** Evaluation of the learning times and the testing accuracies among algorithms using BPNN with 10 principle components

Learning rate *η*	The number of hidden neurons	10	20	30	40	50	60	70	80	90	100
0.0005	Training time (seconds)	4092.19	4213.25	4276.55	4424.77	4455.69	4589.70	4553.02	4640.63	4745.16	4884.30
	Testing accuracy (%)	94.77	94.00	94.16	95.32	94.74	92.62	92.52	90.28	88.77	89.91
0.001	Training time (seconds)	4058.33	4157.41	4218.09	4354.41	4408.23	4500.06	5.75*	1532.38*	2.38*	32.61*
	Testing accuracy (%)	95.02	94.45	95.83	95.51	93.56	91.30	78.17	78.17	78.17	78.17
0.002	Training time (seconds)	**4054.50**	4212.85	4213.71	4385.40	1.11*	1.12*	1.12*	3.44*	1.17*	144.12*
	Testing accuracy (%)	**96.42**	95.72	89.37	86.15	78.17	78.17	78.17	78.17	78.17	78.17

The bold characters represent the structure of the network which shows the best accuracy. The asterisk refers the early termination of the learning process, because the decrement of the error is very small.

**Table 5:** Evaluation of the learning times and the testing accuracies among algorithms using RBFN with 10 principle components

The number of hidden neurons	200	400	600	800	1000	1200	1400	1600	1800	2000
Training time (seconds)	46.03	220.78	290.30	392.64	461.19	852.83	**972.56**	1426.38	1208.97	2008.20
Testing accuracy (%)	86.00	87.81	88.61	88.24	87.40	89.16	**89.36**	89.31	89.21	89.37

The bold column represents the structure of the network which shows the best accuracy.

**Table 6:** Evaluation of the learning times and the testing accuracies among algorithms using SVM with 10 principle components

*C*	1	1000	2000	3000	4000	5000	6000	7000	8000	9000
Training time (seconds)	98.95	52.73	46.83	47.67	39.73	50.84	40.25	37.78	45.89	**39.03**
Testing accuracy (%)	88.89	97.71	98.03	98.19	98.28	98.35	98.39	98.40	98.46	**98.48**

The bold column represents the structure of the network which shows the best accuracy.

In the algorithm based on RBF, we evaluated by varying the number of hidden neurons from 200 to 2000. The accuracy converged to 89% and showed no more improvement after the number of hidden neurons reached 1200. The spread of RBF was chosen by the normalization as Equation (11). The performance of SVM is based on the type of kernel and *C *which controls a weight between the size of margin and the number of misclassified data. We selected the Gaussian kernel like RBF and the sigma of Gaussian kernel is fixed as 2. The *C *was varied between 1 and 9000, and the accuracy converged to 98% when *C *is over 2000.

## Discussion

We evaluated the proposed algorithm in the pre-processing (filtering), feature extraction and classification parts. The proposed algorithm adapted MF as the preprocessing part. 4 ECG descriptive features and morphology features compressed by PCA made up the feature vectors. Since MF is superior in performance to frequency based filtering in terms of preserving ECG morphology, it is thought that MF will become an attractive method concerning the ECG arrhythmia classification algorithm. The distribution characteristics of the proposed ECG descriptive features, especially RRIR and some PCs, showed that they contained effective information.

The proposed algorithm represented the advantages of a fast learning speed and high accuracy in comparison with other gradient based learning algorithms, and was evaluated as having a large dataset. The ELM based algorithm showed the most accurate performance, the SVM based one was slightly lower, but the BPNN and RBF based algorithms were fairly low in terms of accuracy based on when the proposed algorithm and other classifiers based algorithms reached the maximum accuracy with 10 principal components. But the learning time of the proposed algorithm was shorter about 290, 70, and 3 times than that of BPNN, RBF, and SVM based algorithm.

These results demonstrate that the proposed algorithm used more hidden neurons than did the BPNN based algorithm. It reflected the characteristic of ELM that used a portion of the hidden neurons among the randomly setting weights between the input layer and the hidden layer. In addition, we found a tendency for performance to decrease when using over 1800 hidden neurons. This phenomenon was caused by the minimum norm least-squares solution of the linear system in Equation (7) through the Moor-Penrose Generalized Inverse of ***H***. The minimum norm least-squares solution searches for the least-squares solution first, and then searches for the minimum norm among those solutions Therefore, the accuracy of the algorithm concerning the training dataset increases as the number of hidden neurons increases, but the generalization performance decreases when too many hidden neurons are used. Nevertheless, ELM is much easier to decide and evaluate the structure of neural network than BPNN.

The comparisons with other research projects and proposed algorithms, in terms of the size of the training dataset, test dataset and accuracy, are reported in Table 7. The proposed algorithm shows a better performance than those of other existing research projects which were evaluated based on a similar database size. The research projects of Wavelet-PNN7 present higher accuracy results than the proposed one, but it was not performed on the entire MIT-BIH database. In comparison with our earlier work, we have more stable results from a larger database by using MF. The results of the proposed algorithm showed 98.00% in terms of average sensitivity, 97.95% in terms of average specificity, and 98.72% in terms of average accuracy, which was calculated by the weighted average sum of each beat type.

**Table 7:** Comparing the results of preceding research

Method	Number of beat types Training Data Set/Test Data Set	Accuracy (%)
ICA-BPNN[2]	8 (4900/4900)	98.37
DWT-NN[4]	13 (30293/75130)	96.79
FTNN [5]	3 (540/250)	98.0
Wavelet-PNN[6]	6 (11600/11600)	99.65
MOE [21]	4 about (10000/49260)	94.0
Fhyb-HOSA[22]	7 (4035/3150)	96.06
BSS-Fourier[23]	5 (320/160)*	85.04
PCA-ELM[24]	7 (3450/3450)	97.45
Proposed Algorithm	6 (10000/75853)	98.72

* [23] did not use with MIT-BIH arrhythmia DB as the training and test data set.

## Conclusion

We proposed an arrhythmia classification algorithm using ELM in ECG. The proposed algorithm showed effective accuracy performance with a short learning time. In addition, we ascertained the robustness of the proposed algorithm by evaluating the entire MIT-BIH arrhythmia database. All beat types were classified with high accuracy, but in APB the sensitivity was slightly lower than 90%. This result was due to the characteristic of one patient in the MIT-BIH arrhythmia database who had bradycardia and APB at the same time. Thus, the features of that patient's APB beat are similar to the features of other patients' normal beats. Our future direction is to develop a feature set to manage various situations like the one mentioned above, and to separate the subjects included in the training dataset or in the test dataset for better generalization performance.

## Competing interests

The authors declare that they have no competing interests.

## Authors' contributions

JK conceived the study, implemented the algorithm and drafted the manuscript. HS and KS participated in the design and coordination of the study. HS, KS and ML helped analyze and interpret the results, and critically reviewed the manuscript. All authors read and approved the final manuscript.
